# Spiers Memorial Lecture: NMR crystallography

**DOI:** 10.1039/d4fd00151f

**Published:** 2024-09-06

**Authors:** Lyndon Emsley

**Affiliations:** a Institut des Sciences et Ingénierie Chimiques, École Polytechnique Fédérale de Lausanne (EPFL) CH-1015 Lausanne Switzerland lyndon.emsley@epfl.ch

## Abstract

Chemical function is directly related to the spatial arrangement of atoms. Consequently, the determination of atomic-level three-dimensional structures has transformed molecular and materials science over the past 60 years. In this context, solid-state NMR has emerged to become the method of choice for atomic-level characterization of complex materials in powder form. In the following we present an overview of current methods for chemical shift driven NMR crystallography, illustrated with applications to complex materials.

## Abstract 2

We present an overview of current methods for chemical shift driven NMR crystallography, illustrated with applications to complex materials.

## Graphical abstract

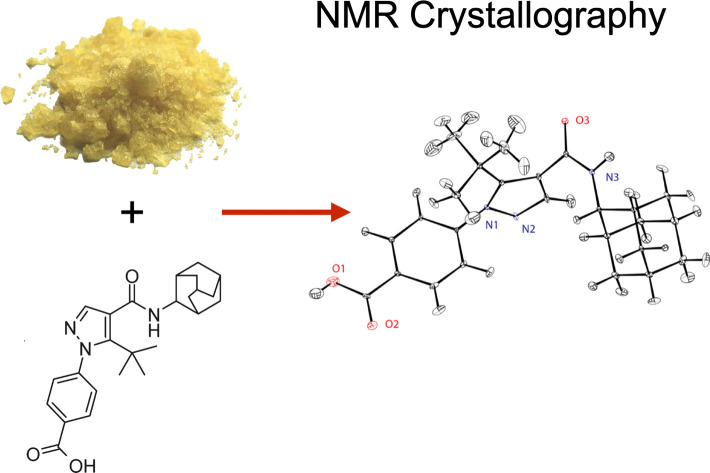

## Introduction

Chemical function is directly related to the spatial arrangement of atoms. Consequently, the determination of atomic-level three-dimensional structures, through the introduction of a range of physical methods, and most notably from single crystals by diffraction methods, has transformed molecular and materials science over the past 60 years, leading to today’s structure-based understanding of chemistry.

Despite this revolution, if the system under investigation is not periodic, or is located on a surface or at an interface, as in many functional materials, or quite simply if it presents in powder form, atomic-level three-dimensional structure determination remains a challenge. Archetypal examples are devices for solar energy conversion, drug formulations, nanoparticles, or cements. As one example, the atomic-level structures at the surfaces and interfaces in hybrid perovskite materials are thought to define the mechanisms that lead to both stabilisation and efficiency of the photo-active phases.^1–4^

In this context, solid-state NMR spectroscopy (in conjunction with diffraction methods and other spectroscopies) has emerged to become the method of choice for atomic-level characterization of complex materials.^5^ Many groups have developed NMR in this direction with notable recent successes in for example MOFs,^6^ cements,^7^ organic semiconductors,^8^ biomass,^9^ battery science,^10^ catalysis,^11^ and hybrid perovskites.^12^ These studies illustrate the very broad impact that NMR can have in materials chemistry when it can be deployed.

In the following, we will give a brief summary of how NMR crystallography is deployed today for powders, and describe some of the most recent developments and applications to complex materials.

## Chemical shift driven NMR powder crystallography

In contrast to methods such as powder XRD^13–15^ or electron diffraction,^16–21^ NMR directly probes the local atomic environment, allowing for structural characterization without the need for long-range order. In this direction, solid-state NMR has seen spectacular progress in the last few years,^5,22–25^ and methods have been introduced to solve crystal structures of bulk inorganic^23,26–34^ or molecular solids.^22,23,35–60^ If isotopic enrichment is possible, NMR methods can be used that lead to complete structures, which is now well established for proteins,^61–68^ and with a more limited set of examples for materials.^27,69–73^

In most cases for organic solids and materials, isotopic labeling can be extremely costly, or the very use of a different synthetic procedure will denature the question. For molecular solids at natural isotopic abundance, methods using proton spin diffusion^35,36,38^ and dipolar recoupling techniques^42,44–48,74,75^ have nevertheless been introduced, and with the demonstration that the sensitivity gains from DNP enhanced methods could yield efficient natural abundance correlation spectra,^76–78^ it has been shown that quantitative carbon–carbon dipolar recoupling experiments are possible at natural abundance.^79–82^

However, in contrast to dipolar couplings, the chemical shift is usually by far the easiest NMR parameter to measure. Together with the development of accurate density functional theory (DFT) based methods to calculate chemical shifts,^83–90^ this has enabled the development of methods using chemical shifts to determine structure (often referred to as NMR crystallography).^39,40,50,89,91–96^ Measured chemical shifts can be compared with computational predictions, and with the current best functionals the average deviation between experiment and calculation can be as low as 1.5 ppm for carbon-13.^22,89,97–101^ This has now been used by many groups to validate or refine crystal structures, with the following being illustrative references.^22,23,36,38,42,44–48,50,51,74,102–134^ The reader is referred to, for example, the article by Brown and coworkers^129^ in this volume for a description of some of these applications. NMR crystallography has been recognized by the International Union of Crystallographers, and chemical shifts are increasingly combined with X-ray or electron diffraction measurements.^60,94,135–150^ Since the first *de novo* chemical shift based structure of a molecular solid solved in 2013,^41^ the technique has been developed and applied to a range of structures,^5,22,23^ from pharmaceuticals^94,113,150–156^ to a metal-binding site in a metalloprotein,^157^ capping groups on nanoparticle surfaces,^95^ the atomic-level structures of calcium silicate hydrates^7,158,159^ or to the spacer layers in two-dimensional hybrid perovskite materials.^160^

Remarkable examples include the determination of the structures of drug molecules in pharmaceutical formulations,^127,161,162^ the detailed determination of the structure of active sites in enzyme reaction pathways,^59,96,163^ or the precise determination of the disordered structure of amorphous drugs.^54,164,165^

**Fig. 1:**
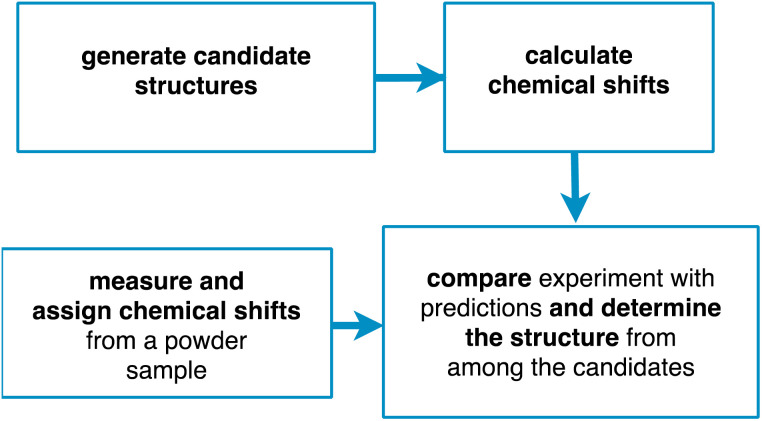
A scheme of the principal steps involved in chemical shift driven NMR crystallography.

Chemical shift driven NMR crystallography usually follows the general scheme involving the four steps shown in Fig. 1. Obviously, the core of the method relies on the experimental measurement and assignment of NMR chemical shifts. As discussed below, this step can range from straightforward, taking minutes to hours, to highly challenging, taking weeks or months. In parallel to the assignment step, candidate structures are generated, either using computational methods or using prior experimentally determined structures (typically from single crystal X-ray diffraction). Then, chemical shifts are predicted for the candidate structures, typically using either DFT calculations or machine learning models. Finally, the predictions are compared with experiment, and a structure is determined, together with attributes such as positional errors and confidence limits.

In the following we will describe each of these steps in more detail. We will focus on the determination of organic molecular solids using isotropic chemical shifts, but the method can be adapted to include chemical shift anisotropy or to hybrid inorganic materials or oxides,^5,22,23,34^ and we will illustrate the methods described with examples primarily from our group. We will also focus on structure determination, rather than structure validation. While the distinction between the two is not always clear, here we will assume that structure determination does not use any prior structural information, while structure validation most commonly refers to the verification (or not) of a hypothesis derived from a pre-existing experimentally determined crystal structure.

### Candidate structure generation

*De novo* structure determination currently requires first the generation of a large ensemble of credible candidate structures.^39–41^ For crystalline materials this is usually achieved with some form of computational crystal structure prediction (CSP) protocol.^33,166–172^ Methods to predict crystal structures have been developed primarily with the objective of understanding the energy landscape of crystal structures, and were first primarily used as a tool to help ensure that all the important crystal forms of a given drug molecule had been found experimentally. This area has come of age over the last 20 years, and rapid progress continues to be made, as can be seen from the ongoing series of blind tests.^173^ The capacity for CSP has increased to include the larger molecules typically found in molecular drugs, having conformational flexibility and including hydrates and solvates of pharmaceutical sized molecules (and we note that CSP can equally be performed for inorganic/ionic or hybrid structures).

From the experimental structure determination perspective, CSP provides an ideal tool to generate an ensemble of possible candidate structures. Notably, while in prediction type applications, accurately ranking structures within a set according to predicted energy is often considered as critical, nevertheless from the experimental structure determination perspective it does not really matter what the predicted energies are, as long as the experimentally present structure is included in the set.

Our objective is not to provide a detailed review of CSP, which can be found elsewhere,^171^ but we note that further improvements in CSP methods, as they become faster, more accurate, and accessible for larger and larger molecules, will directly lead to consequent improvements in CSP based NMR crystallography.

### Chemical shift predictions

Once candidate structures have been generated, the next step is to compute predicted chemical shifts for the ensemble of candidates.^39–41^

#### Plane-wave density functional theory (DFT) methods

While the principles of computing chemical shifts were introduced in the 1950s,^124,174–176^ the challenge here is that high accuracy is required in order to capture the effects of particular conformations and packing arrangements of the molecular building blocks on the chemical shifts, and to allow the identification of the correct structure among a set of potential candidates based on a comparison between computed and measured chemical shifts.

In this respect, a revolution in solid-state NMR has occurred with the introduction of accurate methods to calculate chemical shifts,^83–85^ in particular using DFT methods developed for periodic systems based on the gauge-including projector augmented wave (GIPAW) approach.^87,88,177^ Using a Perdew–Burke–Ernzerhof (PBE) functional^178^ with a dispersion correction^179^ GIPAW can achieve root-mean-square error (RMSE) between experiment and calculation of ∼2.5 ppm for ^13^C and ∼0.4 ppm for ^1^H.^22,34,39,90,124,180^ This accuracy has been key in enabling the very rapid development of chemical shift based NMR crystallography over the last 20 years.

While GIPAW accuracy is sufficient in many cases, increased accuracy of chemical shift predictions will directly lead to increased discriminating power for structure determination by NMR. As a result, there has been a large volume of research aimed at further improving the accuracy of chemical shift predictions with first principles methods. Most of this activity has been directed to using more accurate functionals, which rapidly becomes very computationally costly in the GIPAW formalism. To avoid this, hybrid or double hybrid functionals have been used in cluster or fragment-based approaches. With such calculations, the RMSE between experiment and calculation can be as low as 1.5 ppm for ^13^C and 0.2 ppm for ^1^H.^89,97–101,181,182^

In general, one should bear in mind that the accuracy ranges given above are averages, and that some structures can lead to significant outliers.^183^ Moreover, note that chemical shift calculations depend very strongly on the model structure being used as input. In this regard, if the input is an experimental X-ray diffraction structure, then at least the ^1^H positions should always be optimized prior to calculating chemical shifts, preferably using the same framework as used for the chemical shift calculations.^124,184^ In some cases it is prudent to compare consistency between the results from structures where only ^1^H positions have been optimized to results where the positions of all the heavy atoms have also been optimized. More generally, for candidate structures that have been generated computationally, thought must be given to the level of theory used for structural optimization as compared to the level of theory for the chemical shift computation.^90,185^

Finally, we note that the methods above all compute structures without accounting for finite temperature, meaning that they will not correctly reproduce temperature dependent shifts. To address this, methods have been introduced using averaging over vibrational modes or over snapshots taken from *ab initio* molecular dynamics simulations^186^ or using path integral molecular dynamics.^99,187,188^ While finite temperature effects are a significant practical issue, these approaches currently remain impractical in most cases due to the computational cost. One promising path forward is to use machine learned potentials and chemical shifts for this purpose.^189^

#### Machine learning models

In summary, DFT based methods generally offer a good tradeoff between accuracy and computational cost for computing chemical shifts in small periodic structures. However, the computational cost of DFT methods severely limits the size of systems accessible, preventing the study of large or disordered systems.

To address this limitation of first principles calculations, in recent years machine-learning models have become popular to bypass intensive quantum-mechanical calculations in many areas of chemistry. Indeed, chemical shifts were first predicted in solutions from large experimental databases, and machine-learned models of experimental shifts have met with considerable success, in both small molecules and proteins,^190–199^ and are widely used today.

In contrast, for solids there are no equivalent experimental databases that would be large enough to train prediction models. As an alternative to training models on experimental data, machine-learning models can be built using databases constructed using DFT methods,^200,201^ and this has been applied for chemical shifts in isolated molecules.^200,202–206^ For chemical shifts in solids, an early example of this approach was demonstrated for the specific case of silicas,^207^ and more recently ML models have been developed for molecular solids^58,208–210^ and oxides.^211,212^

Such approaches have proven able to yield chemical shifts with accuracy similar to DFT at a fraction of the computational cost, allowing applications to large ensembles of large systems.

**Fig. 2:**
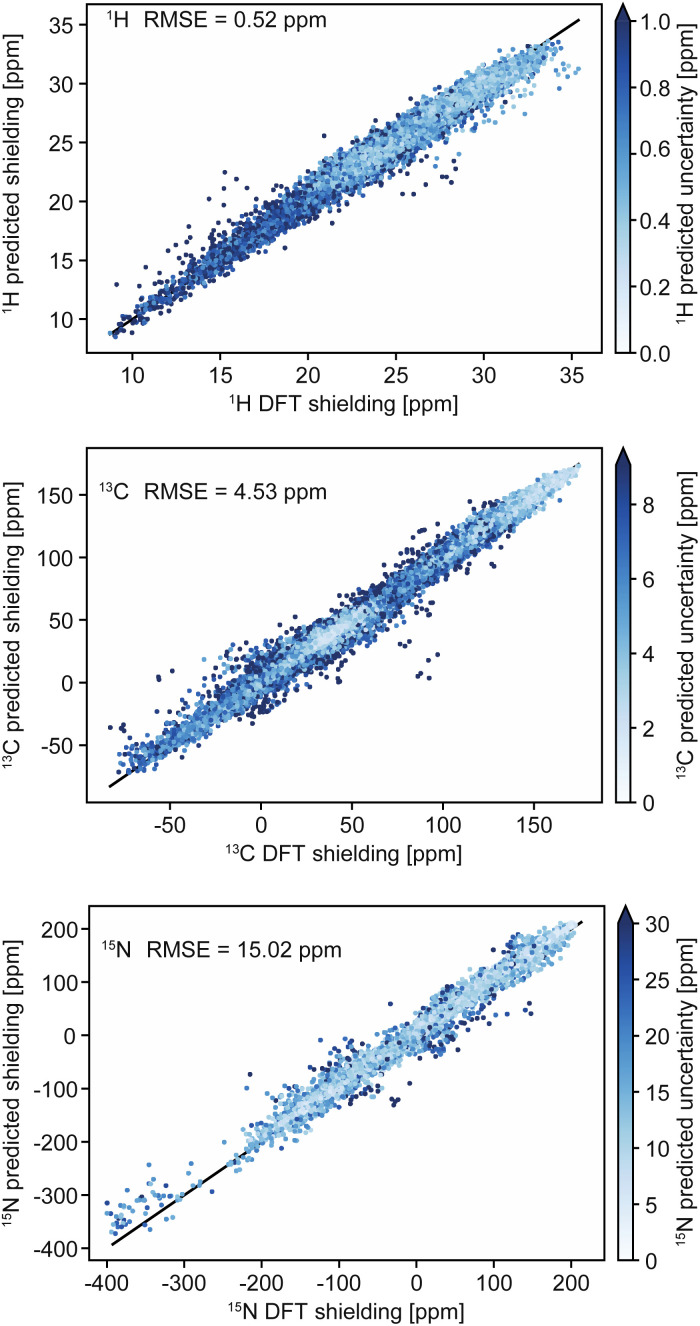
Comparison of DFT-computed shieldings with predictions from the ShiftML2 model for ^1^H, ^13^C and ^15^N. The color scale shows the predicted uncertainty of the ShiftML2 prediction. Adapted from ref. 208.

Chemical shifts in molecular solids present a particular challenge because of the diversity of organic chemistry, and the subtle dependence of shifts on conformations and the effects of crystal packing. In this context we have previously introduced ShiftML,^58^ a machine-learning model of chemical shifts trained on GIPAW DFT data for structures from the Cambridge structural database (CSD).^213^ The current version, ShiftML2,^208^ was trained on GIPAW DFT chemical shifts for an extended set of over 14 000 structures containing any of 12 common elements (H, C, N, O, S, F, P, Cl, Na, Ca, Mg and K), and composed of roughly equal amounts of relaxed and thermally perturbed structures of crystals extracted from the CSD. It allows fast predictions of chemical shifts for any molecular solid containing those atoms with accuracy that is comparable to DFT, and in particular for predictions on distorted structures, or for structures that are geometry optimized using other methods. For example, ShiftML2 yields an RMSE between predicted and experimentally measured ^1^H chemical shifts of 0.47 ppm for a benchmark set of 13 organic molecular solids, as compared to 0.35 ppm using DFT.^208^Fig. 2 shows a comparison of DFT-computed shieldings and predictions with the ShiftML2 model for ^1^H, ^13^C and ^15^N.

### Structure determination

Once candidate structures have been generated together with associated predicted chemical shifts, structure determination is then in principle straightforward by comparison of the predicted shifts with the experimentally determined values.^39,40^

Comparison with experiment is usually done for isotropic ^1^H and ^13^C shifts, but shifts from any other nuclei that are available can be straightforwardly included, most commonly today being ^15^N or ^29^Si, as well as quadrupolar parameters measured from for example, ^35^Cl spectra.^127^ Chemical shift anisotropies (*e.g.*^13^C or ^15^N) can also be compared, when available, although these are usually more laborious to obtain experimentally.

**Fig. 3:**
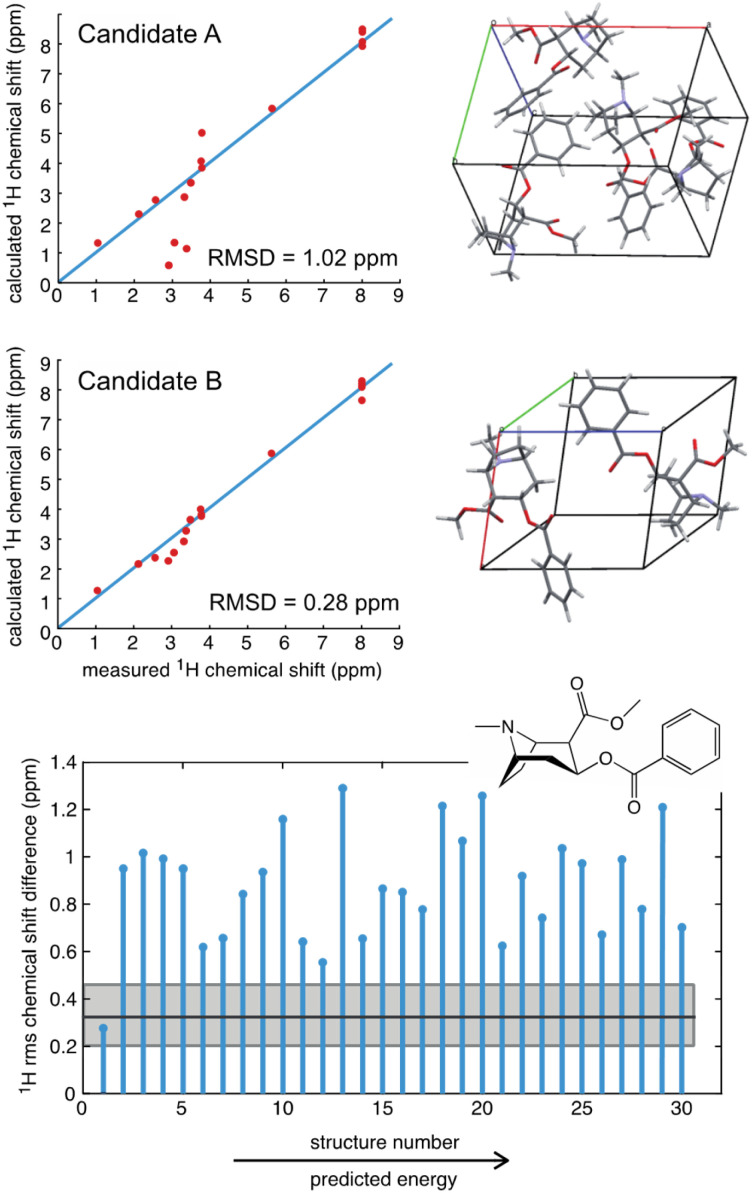
A comparison of the measured ^1^H chemical shifts with predicted values (from GIPAW PBE) for two different candidate structures of cocaine free base (upper), and (lower) a plot of the RMSD for 30 candidate structures.^40^ The solid black horizontal line shows the benchmark mean RMSD error between experimental and predicted shifts, and the horizontal grey shaded zone indicates one standard deviation of the RMSD, such that candidate structures that have an RMSD that falls within the grey zone are in good agreement with the data.

As an example, a comparison of predicted and measured ^1^H shifts for two candidate structures of cocaine are shown in Fig. 3. The agreement between experiment and prediction is usually consolidated into a single value of the average deviation for all the measured chemical shifts, typically expressed as the root mean-squared deviation (RMSD) in ppm,^39,40^ or as a *χ*^2^ value.^214^ In Fig. 3 we can see that the predicted shifts of Candidate A contain some obvious outliers that are not in agreement with the data, leading to an overall ^1^H RMSD of 1.02 ppm. In contrast Candidate B is clearly in better agreement and yields an overall ^1^H RMSD of 0.28 ppm. However, in order to determine if the predicted shifts of either structure are in agreement with the data, there is need to define a benchmark. For molecular solids this was first done for GIPAW PBE calculations using 15 organic compounds where the average RMSD to experiment was found to be 0.33 ppm (±0.16 ppm) for ^1^H and 1.9 ppm (±0.4 ppm) for ^13^C (where the number in brackets corresponds to one standard deviation of the RMSD).^39^ Using this scale, we can see that Candidate A does not agree with the data, whereas Candidate B is agreement with the data to within error.

**Fig. 4:**
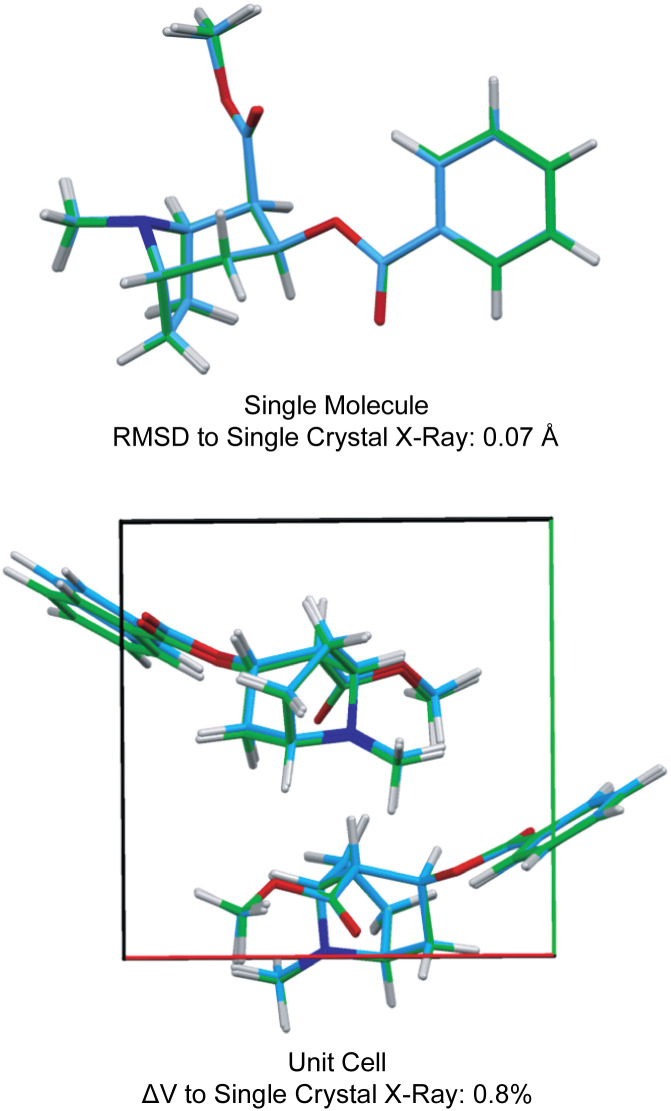
A comparison of the structure of cocaine free base determined by powder ^1^H NMR and the structure determined by single crystal X-ray diffraction. Adapted from ref. 40.

Fig. 3 also shows the RMSD between prediction and experiment plotted for a series of 30 candidate structures generated using a comprehensive CSP protocol, and where the candidates correspond to the set of predictions that have computed energies that are within 10 kJ mol^−1^ of the most stable structure.^40^ It can immediately be seen that of all 30 structures, only one structure is in good agreement with the data. The full crystal structure of cocaine determined in this way from the powder sample is shown in Fig. 4, where it is overlaid with the known structure of cocaine that had been previously determined by X-ray diffraction from a single crystal sample, where they are seen to be essentially identical.

To provide a complete structural picture, two key elements of structure determination are the level of confidence associated with the determined structure, and the estimation of the errors on the positions. To address confidence, Bayesian approaches have been introduced that assign a probabilistic value that quantifies the likelihood that a given candidate corresponds to the experimental structure.^55,214,215^ Using the approach of Engel *et al.*,^55^ the structure of cocaine determined in Fig. 3 using ^1^H shifts has a confidence of 95 or 100%, depending on if predicted shifts from ShiftML1 or from GIPAW are used. The article by Mueller^214^ in this volume provides an excellent overview of the determination of confidence.

We do note that, in general, there is a threshold number of chemical shifts below which there is generally poor confidence in the structure.^55^ This can be explained in the sense that if the molecule only has, *e.g.*, three different types of ^1^H/^13^C, then many candidate structures may accidently agree with the data. However, once the molecule has, *e.g.*, 10 or more shifts, accidental agreement becomes much less likely. A good example of this is theophylline, where 11 out of 45 candidate structures were found to have ^1^H RMSDs <0.5 ppm.^40^ Interestingly, we see that chemical shift driven structure determination will typically work better in larger, more complex, molecules.

Positional uncertainties for the structures obtained by chemical shift driven NMR crystallography can be quantified by estimating the correlation between the chemical shift RMSD and the variances of atomic positions of individual atoms,^57^ thereby making NMR structures directly comparable to structures determined by other methods. This is conveniently achieved by calculating chemical shifts for an ensemble of slightly perturbed crystal structures obtained by MD simulations at finite temperatures, and characterizing the deviation in position that is required to exceed the uncertainty in the chemical shifts.^57^ The positional distributions obtained in this manner are then converted into anisotropic displacement parameters (ADPs), which can be represented by ellipsoids on the determined structure. For the structure of cocaine, this leads to an average positional RMSD <*r*_av_> of 0.169 Å, corresponding to an average equivalent displacement parameter of 0.0095 Å^2^.^57^

It is interesting to note that the positional uncertainties obtained for powder NMR structures are similar to those obtained for single crystal X-ray diffraction structures. Further we note that since the chemical shifts are not reliant on long range order, the positional uncertainty should not change significantly with molecular size. Indeed, Holmes *et al.* have reported average positional RMSD of 0.17 Å for the ^1^H atoms for structures of the co-factor and substrates for the α-aminoacrylate intermediate of tryptophan synthase in the enzyme active sites.^216^

**Fig. 5:**
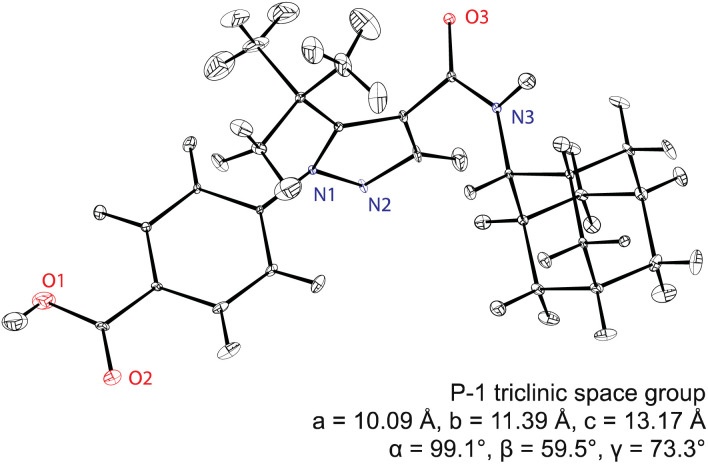
The *de novo* structure of AZD8329 determined by ^1^H chemical shift driven NMR powder crystallography.^55,57,217^ Adapted from ref. 57.

Assembling all the elements described above, Fig. 5 shows the NMR structure of the form 4 of the drug (4-[4-(2-adamantylcarbamoyl)-5-*tert*-butyl-pyrazol-1-yl] benzoic acid) (AZD8329). This was the first example of NMR structure determination for a molecular compound of previously unknown structure. The structure is determined with 100% confidence, and with a positional RMSD of 0.17 Å (corresponding to an average equivalent displacement parameter of 0.0095 Å^2^).

**Fig. 6:**
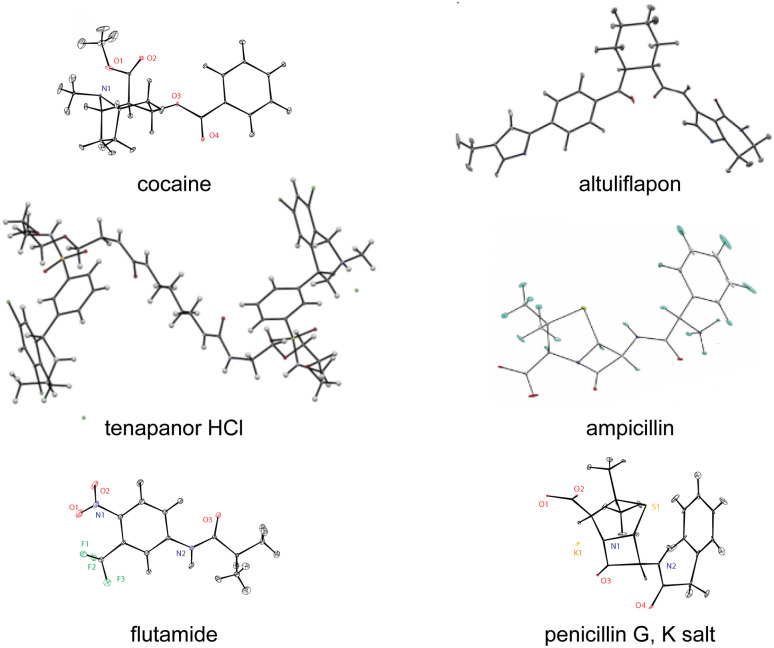
A set of illustrative structures determined from microcrystalline powder samples by chemical shift driven NMR crystallography.^40,54,57,94,218^

There are today a large and growing number of compounds with structures that have been determined by chemical shift driven powder crystallography using the protocols described above. Fig. 6 shows a series of examples from the work of our group. In addition, some other illustrative examples are the structures of a methanol solvate-hydrate of decitabine,^219^ catechin,^151^ furazidine polymorphs,^220^ aspirin,^154^ teriflunomide,^153^ mebendazole,^155^ linezolid cocrystals,^152^ three pyridine dicarboxylic acids,^221^ leucopterin,^150^ and the series of structures from actives sites in tryptophan synthase.^59,96,163^

### Systems lacking long-range order

Because the chemical shift is only typically sensitive to the environment up to a range of 5–8 Å from a given nucleus, chemical shift driven NMR crystallography does not require longer range order in the sample to determine structure. This is particularly striking in the case of amorphous solids discussed below, but in particular it allows the straightforward study of hierarchical, composite or slightly disordered materials. One example of this would be the determination of the structure of an active pharmaceutical ingredient inside a tablet formulation.^162^

**Fig. 7:**
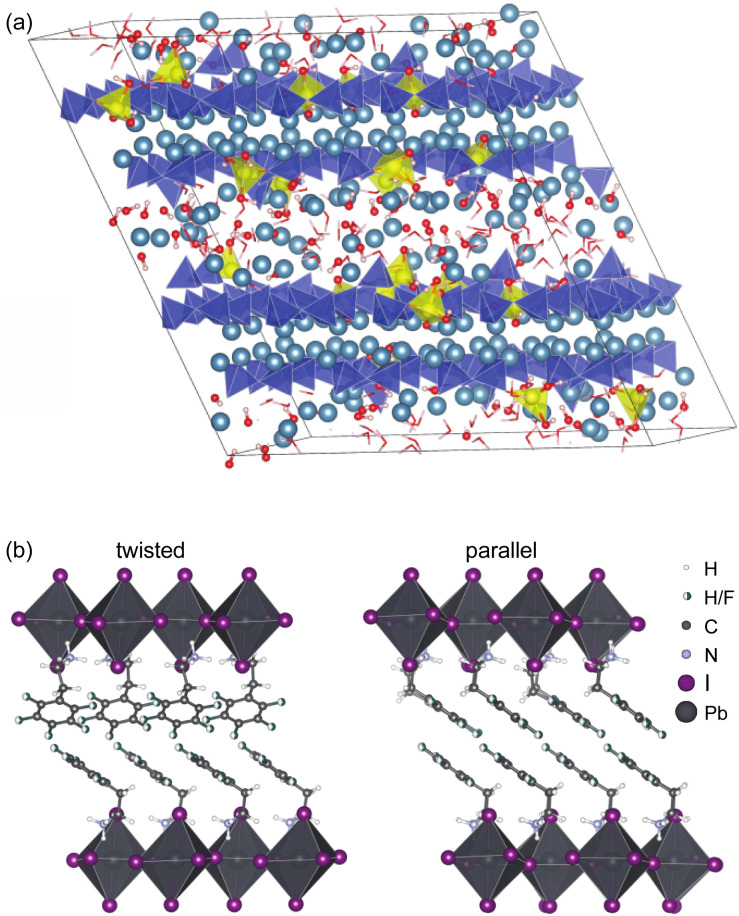
(a) Representative atomic-level structure of zinc-modified C-S-H as determined with a Zn : Si ratio of 0.15. Silicate tetrahedra are depicted in blue; zincate tetrahedra are depicted in yellow; and calcium ions are depicted in light blue,^159^ and (b) the structure of S_2_PbI_4_ layered perovskites with twisted (left) and parallel (right) relative orientations of the aromatic rings in adjacent layers.^160^ Adapted from ref. 159 and 160.

Another illustrative example is the determination of the structure of organic spacer layers contained in layered hybrid lead-halide perovskites,^160,222^ where the inorganic layers are slightly disordered, and where the thickness of the inorganic layers varies from layer to layer. In such systems, although the organic spacer layers as determined by NMR can be highly ordered, as shown in Fig. 7, with two different but well-defined structures forming nano-domains,^160^ these structures would be essentially invisible to diffraction methods due to the overall longer-range disorder in the hierarchy of the structure.

Notably, by providing this atomic-level window on the details of these complex hierarchical and locally disordered structures, the structural information obtained from NMR has guided new strategies for the design and production of new perovskite formulations that yield better performance in terms of both photovoltaic efficiency^12^ and environmental stability.^223^

Another illustration is the determination of the complete atomic-level structures of various calcium silicate hydrates (C-S-H),^7,159,224^ which are the main components of Portland cement. The structure of pure C-S-H determined by NMR is found to correspond to an ensemble of defective tobermorite unit cells including calcium sites in the interlayer that bridge chain-terminating silicate Q^(1)^ sites.^224^ This site is associated with an environment of strong hydrogen bonding, which stabilizes the structure and, consequently, promotes high Ca : Si ratios in C-S-H. The NMR structure establishes a clear relation between the atomic-level defect structure and the high Ca : Si ratio in C-S-H. Similar structures have also been determined for aluminium^7^ and zinc containing C-S-H, with an illustrative Zn–C-S-H structure shown in Fig. 7.^159^ The knowledge of these structures is a prerequisite for overcoming the self-limiting growth of C-S-H and to better understand growth mechanisms and kinetics. Once again, despite the lack of long-range order due to the presence of defects, chemical shifts contain all the information needed to fully characterize the structure.

### Amorphous solids

Another important category of materials lacking long-range order that can be accessed by NMR crystallography is the area of amorphous solids. Indeed there is a long history of the application of NMR spectroscopy to study amorphous inorganic glasses or polymers.^5^ Amorphous molecular solids are becoming increasingly important especially in view of the development of amorphous drug formulations in the pharmaceutical industry.^225–228^ However, while NMR has been used to study some aspects of amorphous compounds,^22^ complete atomic-level structures are required to rationalize the factors that lead to the stabilization of amorphous forms.

The structure determination process for amorphous molecular solids follows the same outline as for crystalline materials in Fig. 1, but with some important differences.^54,164,165^

**Scheme 1:**
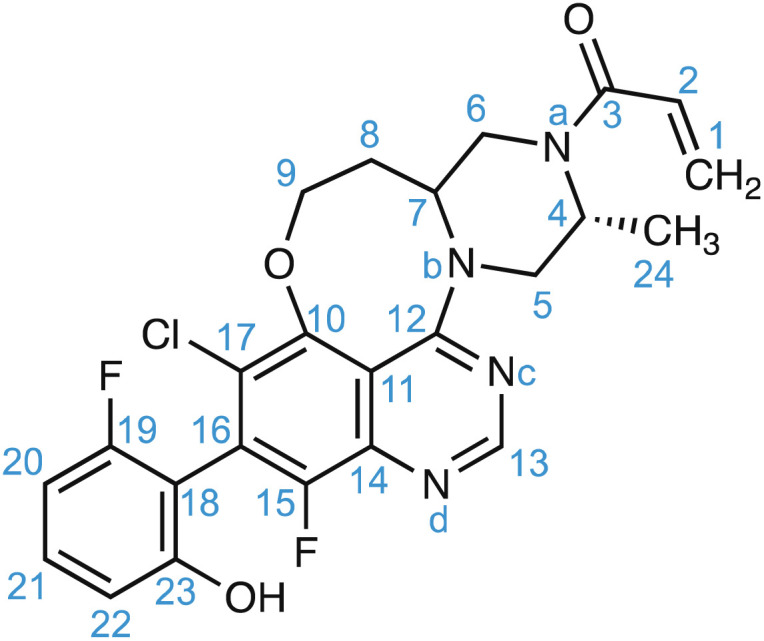
The chemical structure and numbering of AZD4625.

For amorphous solids, structure generation is achieved using molecular dynamics simulations. As an example, for the drug molecule AZD4625 (with the chemical structure shown in Scheme 1), eight MD simulations were carried out with cells containing 128 molecules of AZD4625, randomly initialized in order to model an amorphous system. Chemical shift predictions were then performed using ShiftML2 for 8000 snapshots taken from the MD trajectories, corresponding to more than 1 million molecules. The predicted shifts were then compared with the experimental values obtained for ^1^H and ^13^C for all the molecular environments extracted from the MD snapshots, where each environment comprises a central molecule and all molecules with at least one atom within 7 Å from any atom of the central molecule. For each atomic site, the probability that the predicted shift is drawn from the corresponding experimental chemical shift distribution was calculated and combined into a global probability that the molecular environment matches the NMR experiments. Then the top 1% of local molecular environments in best agreement with experiment were taken as a set to describe the experimental structure, referred to as the NMR set. More details are given in ref. 165. A similar approach was used for the drug Atuliflapon.^54,164^ It is worth pointing out that care should be taken to make sure that the range of conformations generated as candidates by the molecular dynamics simulations is larger than those present in the sample, and to assess the presence of any actual molecular dynamics that might be present in the samples which could affect the observed lines’ shapes.^229^ This latter point is common to any NMR crystallography investigation, whether on crystalline or disordered samples.^5^

**Fig. 8:**
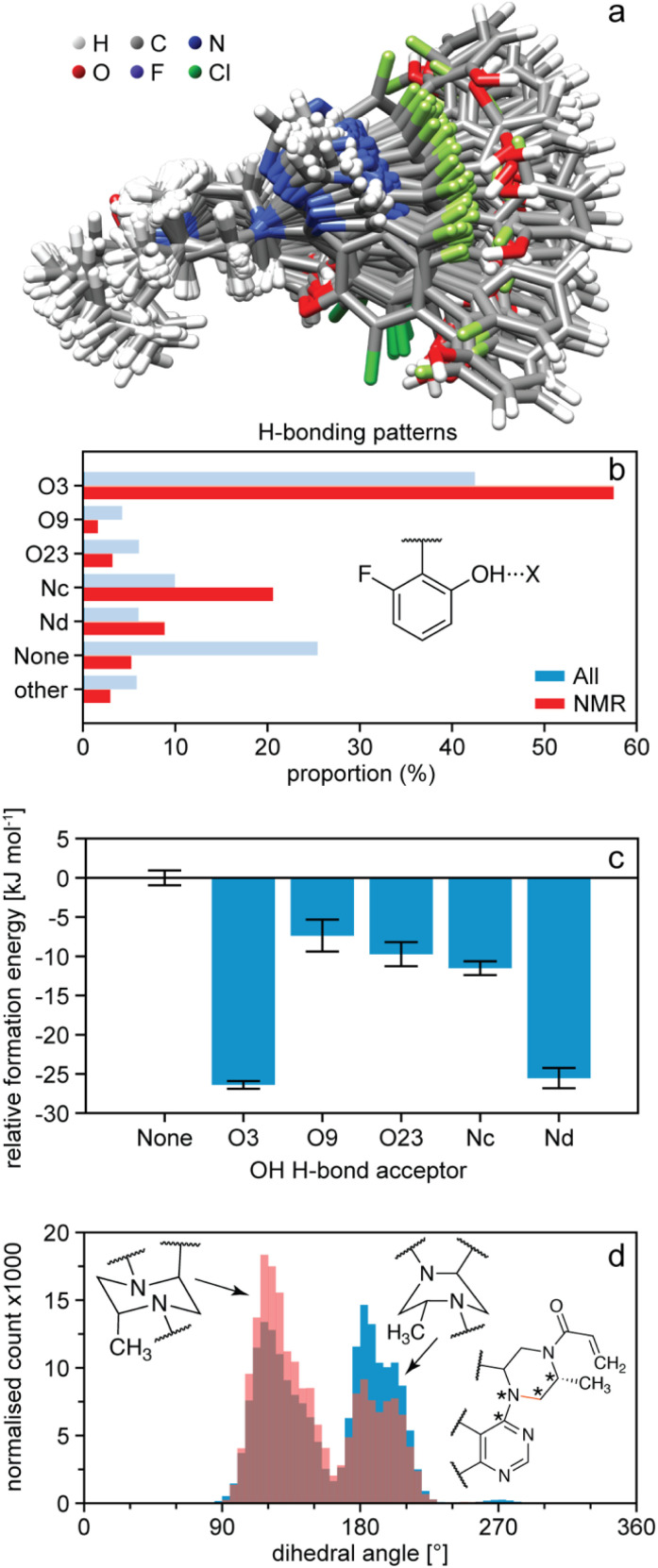
The structure of amorphous AZD4625.^165^ (a) Superposition of 100 conformations randomly sampled from the NMR set, and aligned on atoms Nb–C5–C4–Na. (b) Proportions of different hydrogen bond acceptors bonded to the OH group of AZD4625 in all local molecular environments (blue) and in the NMR set (red). (c) Computed relative formation energies of the local molecular environments in the NMR set for different hydrogen bond acceptors bonded to the OH proton. The zero is set to be the mean formation energy of intermolecular complexes where no hydrogen bonding acceptor is bonded to the OH proton of the central molecule. (d) Histogram of dihedral angles for the aliphatic ring in all molecules (blue) and in the NMR set (pink). The rotatable bond associated with the dihedral angle is drawn in orange. Stars indicate the atoms used for the computation of the dihedral angle. Adapted from ref. 165.

Once the NMR set has been obtained, it can be analysed in terms of the distributions of structural features present in the amorphous structure and correlated to predicted energies.^54,164,165^ For example, Fig. 8b shows the occurrence of hydrogen bonding patterns observed in the NMR set of AZD4625 as compared to the background MD set.^165^ Notably, over 25% of environments in the MD set have no hydrogen bond to the OH proton, whereas almost all the environments are H-bonded in the NMR structure, with a particularly strong promotion of H-bonding to O3. This can be directly correlated to the computed energies shown in Fig. 8c, where a clear stabilisation of the structure by the O3 H-bonding interaction is observed. Conversely, it is interesting to note that H-bonding to Nd is predicted to be strongly stabilising, but that this is not significantly promoted in the experimental structure. Similarly, the structure can be analysed in terms of preferred conformations. Fig. 8d shows the population of conformations of the aliphatic ring in AZD4625, which shows a clear preference for the chair conformation in the experimental structure. A similar analysis has been carried out for Atuliflapon.^164^

### Direct structure optimization

**Fig. 9:**
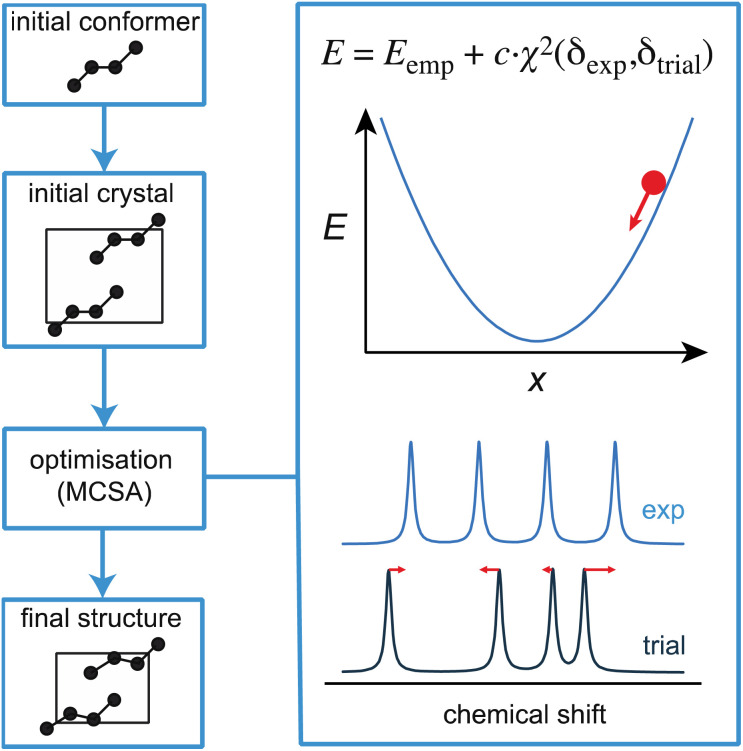
A schematic outline of one approach to direct structural optimisation using on-the-fly chemical shifts. The driving force for optimisation is an empirical energy term that is the sum of a computed internal energy term *E* that ensures physically realistic structures, and a term that drives agreement between experimental and predicted chemical shifts.^218^

Before moving on to discuss experimental approaches to measuring and assigning chemical shifts, we note that there is ample room for improvement in the structure generation and shift prediction steps for NMR crystallography, and this is an active area of research. For example, while powerful, CSP is a time-consuming approach whose efficiency could be greatly improved by making use of chemical shifts at an earlier stage of the process. Moreover, if the set of candidates does not contain the correct structure, then the process fails, and in these cases requires the addition of some knowledge of internuclear distances to constrain or direct the CSP process.^56,81,92^ In contrast, more established approaches to *de novo* structure determination, for example by single-crystal X-ray diffraction of large molecules or by solution NMR, usually involve an iterative process where a (often random) starting structure is optimized under the combined effect of an (usually empirical) energetic potential and a penalty term that compares the computed observables with the measured values at every step of the optimization.^230^ This is a very powerful approach to finding the correct structure, and is enabled by the fact that the calculation of observables from any trial structure is rapid. Until recently, this had not been possible in chemical shift based NMR crystallography, except for a few exceptions where shifts could be derived from force-fields,^231,232^ since it would have required the DFT calculations discussed above. We have recently shown how the structure of microcrystalline molecular solids can be determined by integrating on-the-fly shift calculations using ShiftML into a simulated annealing optimization protocol, as illustrated in Fig. 9.^218^ The approach was demonstrated to successfully determine five crystal structures, and notably for two different polymorphs of the drug molecule AZD8329.^218^ While this proof-of-concept implementation is exciting, we note that this approach is not as straightforward as it seems, since it requires high accuracy in shift predictions and because optimizing crystal structures is very different from optimizing isolated molecules.

### Measuring and assigning chemical shifts

We now turn our attention to the experimental measurement and assignment of chemical shifts in powdered molecular solids at natural isotopic abundance. This is a prerequisite for any NMR crystallography study, and has been the subject of intense ongoing research for the last 80 years, with hundreds of different NMR pulse sequences having been developed and tested.^5^ We will not review this large body of work here, but rather propose to illustrate the two main approaches used to assign ^1^H, ^13^C and ^15^N spectra in molecular solids at natural isotopic abundance today, with illustrations from our own group.

First, in general, all of the state-of-the-art assignment methods are based on using magic angle spinning (MAS)^233,234^ to obtain high-resolution NMR spectra. Spectral resolution is then further increased by acquisition of two (or higher) dimensional correlation spectra, and the nature of the correlations is then used to determine site-specific assignments of the observed resonance frequencies to each of the nuclei in the molecule/material. Correlations are most often obtained using cross polarization^235^ to transfer magnetization from one nuclei to another *via* the through-space dipolar coupling.

**Fig. 10:**
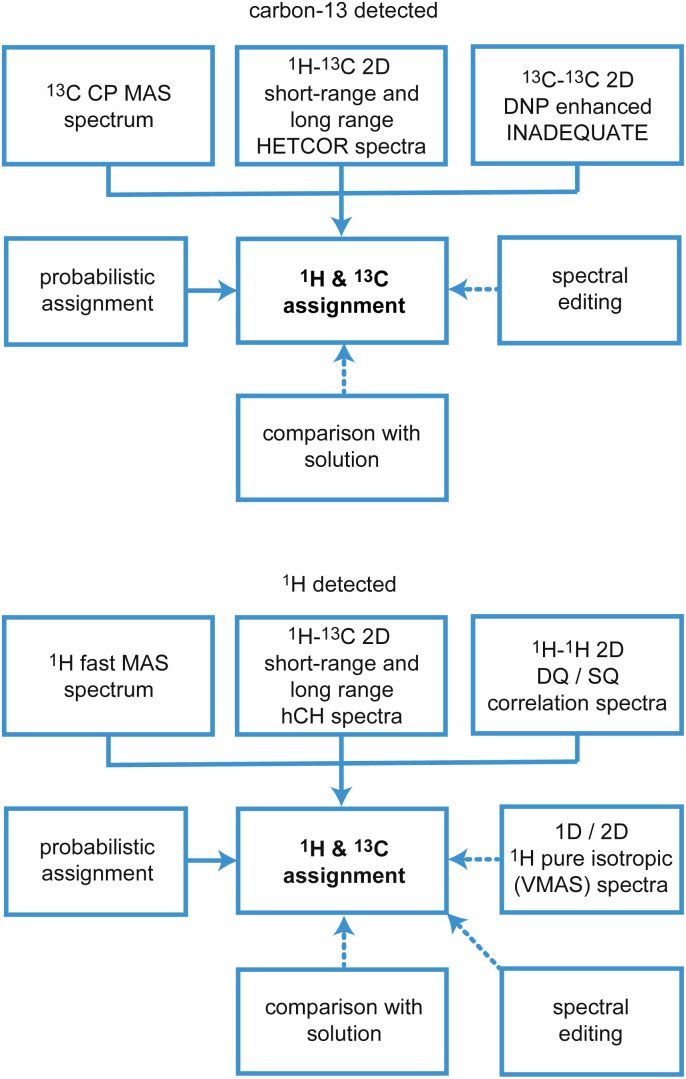
Schematic workflows for assignment using ^13^C (upper) and ^1^H (lower) detection strategies. Solid arrows indicate core steps in the assignment process that are always used, while dotted arrows indicate the most typical supplementary experiments and approaches that are used in cases where the core techniques leave some ambiguity.

There are two main approaches to implement this, which are based on (i) carbon-13 (or ^15^N, ^29^Si, ^31^P…), or (ii) proton detected experiments, and the schematic workflows are illustrated in Fig. 10.

### Assignment using carbon-13 detected experiments

**Fig. 11:**
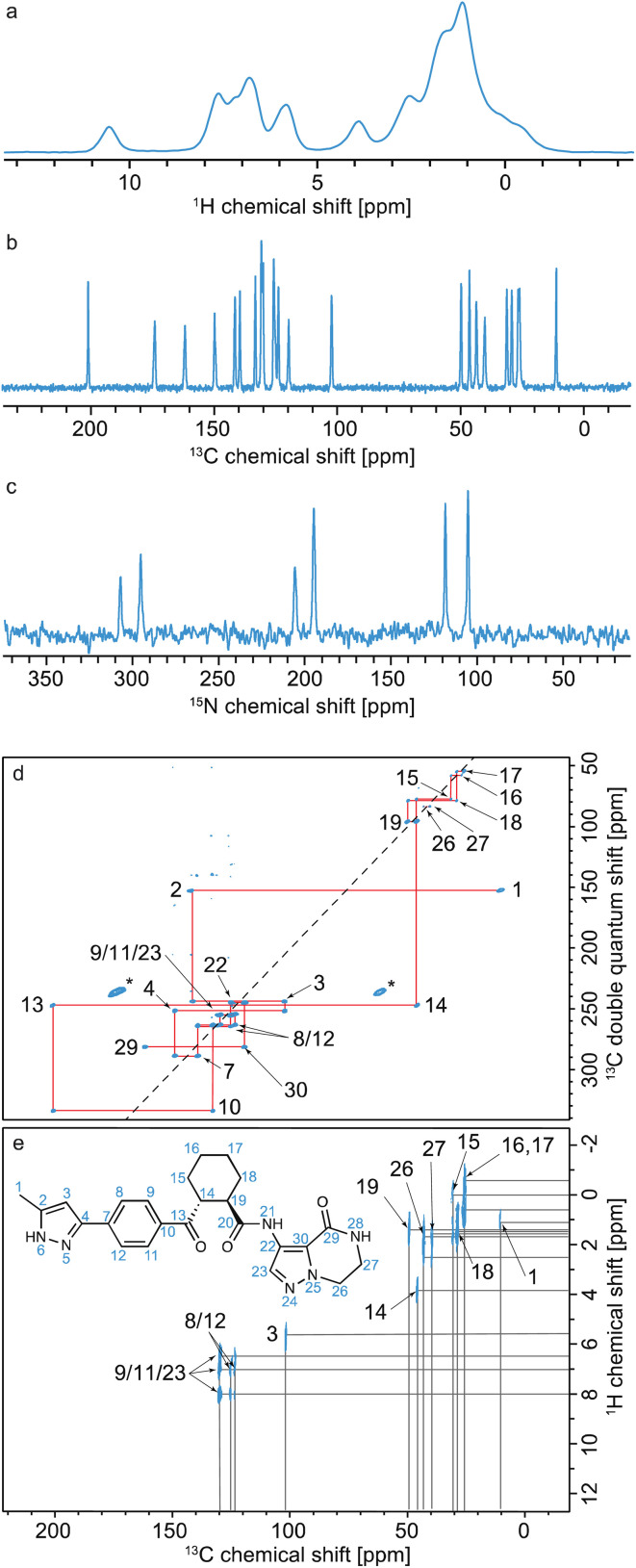
Spectra used for the carbon-13 detection based assignment of Atuliflapon (chemical structure shown inset).^54^ One dimensional (a) ^1^H (21.1 T, 111 kHz MAS rate, 298 K), (b) ^13^C CP (11.7 T, 22 kHz MAS rate, 298 K) and (c) ^15^N CP (9.4 T, 12 kHz MAS rate, 298 K) NMR spectra. (d) ^13^C–^13^C DNP enhanced solvent suppressed INADEQUATE (9.4 T, 12.5 kHz MAS rate, 100 K), and (e), ^1^H–^13^C HETCOR spectra (11.7 T, 22 kHz MAS rate, with eDUMBO-1_22_ homonuclear decoupling^236^ in *t*_1_). In (d), the ^13^C peaks denoted by an asterisk at 60 and 170 ppm are attributed to impurities introduced during sample preparation. Adapted from ref. 54.

Typically, as shown in Fig. 10, all assignment strategies start with the acquisition of one-dimensional ^1^H directly detected and ^13^C CPMAS spectra. Examples of these are shown in Fig. 11 for the example of a sample of micro-crystalline Atuliflapon,^54^ together with the one-dimensional ^15^N CPMAS spectrum.

In both strategies, the core methods for assignment involve two-dimensional correlation spectra, and the approaches diverge in the set of spectra that are used. For ^13^C detected experiments, the two workhorse correlation experiments used for assignment are ^13^C detected ^1^H–^13^C HETCOR^5,237,238^ and ^13^C–^13^C refocused INADEQUATE^239^ spectra. Spectra are usually acquired using 3–4 mm rotors, with MAS rates of 8–12 kHz.

HETCOR spectra correlate ^1^H and ^13^C spectra through cross-polarization from ^1^H to ^13^C, followed by detection of the ^13^C signal. This yields dipolar mediated (*i.e.* through-space) correlations between the isotropic chemical shifts of protons in *ω*_1_, and isotropic chemical shifts of ^13^C in *ω*_2_. Typically, two HETCOR spectra are recorded; one with a short (*e.g.* 100 μs for the spectrum shown in Fig. 11e) CP contact time that yields correlations primarily between directly bonded nuclei, and a second with a longer (*e.g.* 4 ms) CP contact time that yields correlations with nuclei that are up to around 3–5 Å apart.

Refocused INADEQUATE spectra yield *J*-mediated (*i.e.* through-bond) correlations between directly-bonded pairs of carbon-13 nuclei, as illustrated in the spectrum of Fig. 11d.

Armed with these two spectra, complete ^1^H and ^13^C assignment can often be directly achieved by first mapping out all the connections between bonded ^13^C seen in the refocused INADEQUATE spectrum, and then connecting each of the assigned ^13^C resonances to their attached proton. This is the case for Atuliflapon using the spectra shown in Fig. 11.

However, in many cases, spectral overlap or missing correlations may render parts of the assignment ambiguous, and these two correlation spectra alone may not be sufficient. In that case, there are two straightforward additions that are used. First, spectral editing techniques^240–243^ can be used to simplify the one-dimensional ^13^C spectra and/or the HETCOR spectra and assign a multiplicity to each of the ^13^C resonances.

**Fig. 12:**
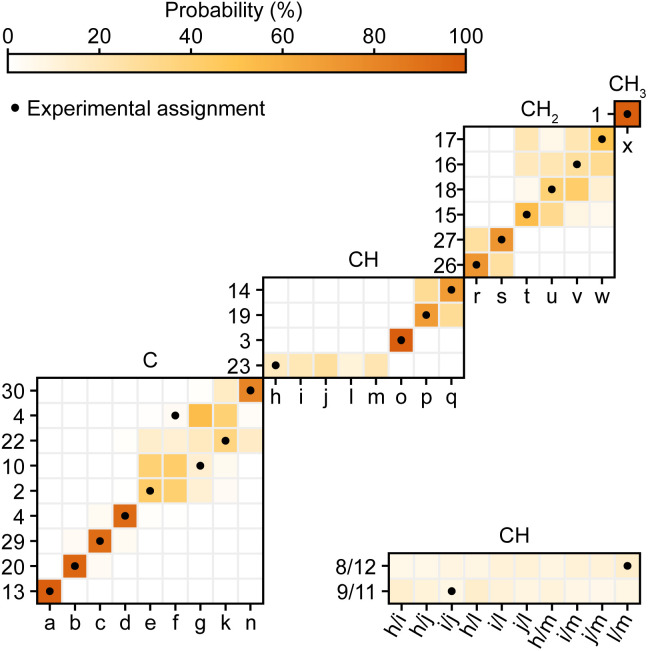
Marginal individual assignment probabilities of ^13^C chemical shifts of Atuliflapon obtained using correlated ^1^H–^13^C chemical shift distributions and spectral editing.^244^ For the probability map, labels along the vertical axis indicate nuclei (according to the inset in Fig. 11), and labels along the horizontal axis denote experimental shifts labelled alphabetically in order of decreasing ^13^C shift. The carbon multiplicity is indicated above each marginal assignment probability map. Adapted from ref. 244.

Second, Cordova *et al.*^244^ recently developed a method for automated probabilistic assignment of experimental chemical shifts for molecular solids directly from their two-dimensional molecular structure using a statistical analysis of a shift database that was constructed by computing shifts for over 200 000 compounds in the Cambridge Structural Database using ShiftML. An example of this applied to Atuliflapon is shown in Fig. 12 where we see that most of the chemical shifts are directly assigned with high levels of confidence, typically leaving only a few permutations of pairs or triples that would need to be resolved by analysis of the two-dimensional connectivities.^244^ This probabilistic method has no extra cost in terms of experimental acquisition, and when applied to 2D X–H correlation spectra, with or without spectral editing, proves to be very robust. It is now a core guide in all our assignment workflows.

The ^1^H and ^13^C assignment of Ritonavir provides a particularly complete recent example of carbon-13 based assignment using HETCOR, refocused-INADEQUATE, spectral editing and the probabilistic method.^244^

There is one key Achilles’ heel to the carbon-13 based methods, which is sensitivity. The refocused-INADEQUATE experiment correlates pairs of directly bonded carbon-13 nuclei, which only occur every 1 in 10 000 at natural isotopic abundance. While De Paëpe *et al.* showed 20 years ago that it is possible to obtain refocused-INADEQUATE spectra from natural products with up to 30 carbon atoms in favorable cases, if the experiment is carefully optimized and long (∼1 week) acquisition times are used,^245^ in most practical cases for molecules with more than 10 carbon atoms this is not possible.

The sensitivity problem in MAS NMR has in principle been alleviated with the introduction of high-field dynamic nuclear polarization (DNP),^246,247^ which can increase NMR signal intensity by up to two orders of magnitude. MAS DNP has been extensively developed with the introduction of impregnation methods for materials,^76,248,249^ and can today yield signal enhancements of up to a factor 100 for powdered molecular solids. This has had a transformative effect, and in particular with the demonstration that efficient natural abundance ^13^C–^13^C correlation spectra can be obtained, DNP^76–78^ has made the carbon-13 based assignment strategy outlined above practical. Indeed, the refocused-INADEQUATE spectrum shown in Fig. 11d was only accessible with the use of DNP.

That said, enabling DNP requires formulating the sample with a non-solvent containing a polarizing agent, and finding a formulation that yields significant DNP enhancements on the target substrate is often not simple. If DNP enhancements more than a factor 10 cannot be achieved, natural abundance ^13^C–^13^C correlations are often inaccessible. In turn, the absence of a ^13^C–^13^C correlation makes carbon-13 based assignment extremely challenging in all but the most straightforward cases.

### Assignment using ^1^H detected experiments

Another route to sensitivity enhancement in solid-state NMR would be to adopt ^1^H detection strategies. Indeed solution-state NMR has been driven by ^1^H based detection strategies, primarily enabled by the high spectral resolution obtained in solution-state ^1^H NMR. The more limited use of these methods in solids is because ^1^H NMR spectra of solids are typically two-orders of magnitude less well resolved.^5^ However, in cases where the resolution in the proton spectrum is sufficient, the advantage provided by ^1^H detection in solids is clearly established both in biological applications^5,66,250–257^ and in materials samples,^42,258–276^ including in combination with DNP.^277–282^

The advent of faster MAS, which usually leads to better resolved ^1^H spectra, has been a key factor in enabling ^1^H detection in a broader range of systems.^42,256,275,283–288^ Due to hardware advances, we are now able to reach magic-angle spinning (MAS) rates above 100 kHz. At such spinning rates MAS results in sufficient ^1^H line narrowing for the acquisition of high-resolution 1D and 2D ^1^H-detected spectra for typical molecular solids. For such materials, transitioning to ^1^H detection instead of ^13^C detection translates to faster acquisition due to the high sensitivity of protons, and avoids the need for hyperpolarization by DNP.

**Fig. 13:**
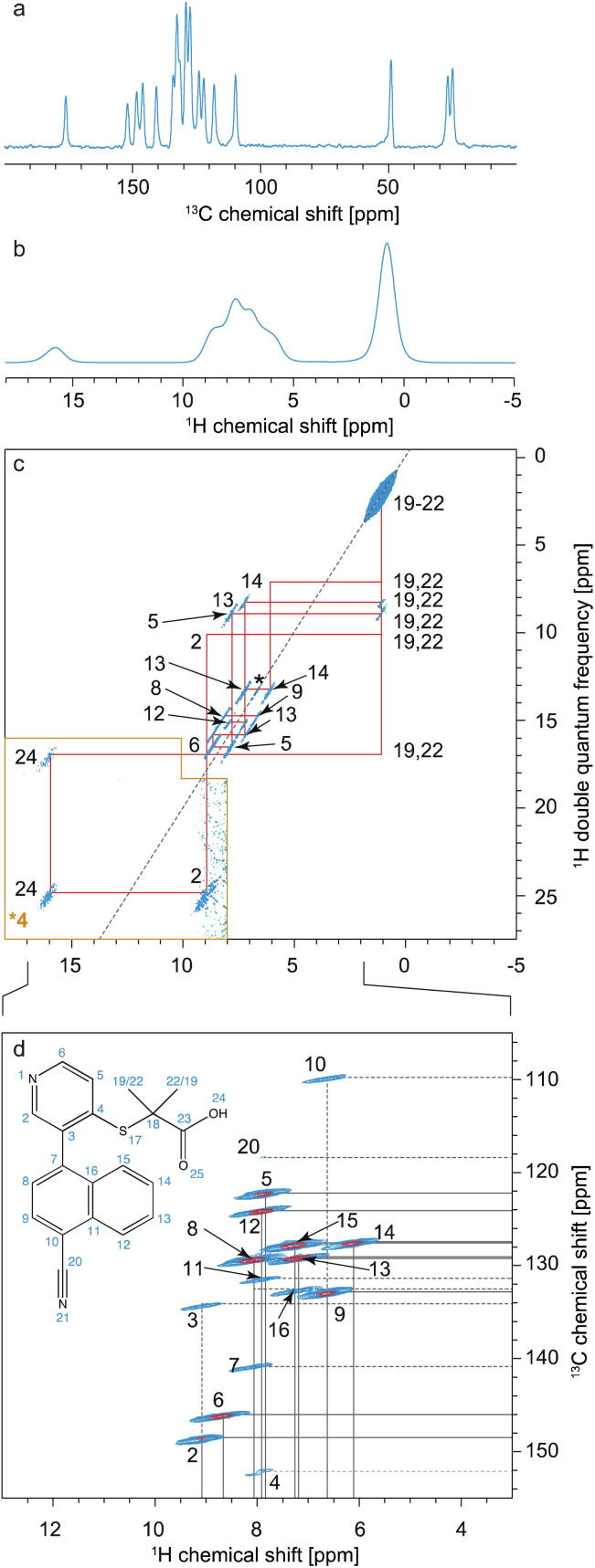
Spectra used for the ^1^H detection based assignment of Verinurad (chemical structure shown inset).^128^ (a) 1D ^1^H spectrum at 160 kHz MAS. (b) ^13^C CP spectrum at 100 kHz MAS. (c) 2D ^1^H–^1^H BABA spectrum obtained at 160 kHz MAS. Red lines indicate the connectivities between cross peaks. (d) The aromatic region of the 2D hCH long-range spectrum in blue (4 ms direct CP contact time) overlaid with a 2D hCH short-range spectrum in red. The assigned cross-peaks are indicated by labels, with solid lines for protonated carbons and dashed lines for quaternary carbons. Adapted from ref. 128.

For ^1^H detected experiments, the two workhorse correlation experiments used for assignment are ^1^H detected hCH^66,268,270,289^ and ^1^H–^1^H double-quantum–single-quantum (DQ/SQ) experiments,^42,290,291^ as illustrated in Fig. 13 for the case of Verinurad.^128^ Spectra are usually acquired using sub 2 mm rotors, with MAS rates >50 kHz.

hCH spectra correlate ^1^H and ^13^C spectra through cross-polarization from ^1^H to ^13^C, followed by evolution of ^13^C magnetization and back CP transfer to ^1^H for detection. This yields dipolar mediated (*i.e.* through-space) correlations between the isotropic chemical shifts of carbon-13 in *ω*_1_, and isotropic chemical shifts of protons in *ω*_2_. Typically, two hCH spectra are recorded; one with a short (*e.g.* 250 μs for the red spectrum shown in Fig. 13d) back CP contact time that yields correlations primarily between directly bonded nuclei, and a second with a longer (*e.g.* 4 ms for the blue spectrum in Fig. 13d) back CP contact time that yields correlations with nuclei that are up to around 3–5 Å apart.

^1^H–^1^H DQ/SQ spectra yield dipolar-mediated (*i.e.* through-space) correlations between pairs of ^1^H nuclei, typically separated by distances up to 5 Å depending on the mixing times used for double-quantum excitation and reconversion, as illustrated in the spectrum of Fig. 13c.

In analogy to the use of the carbon-13 detected spectra above, complete ^1^H and ^13^C assignment can then be directly achieved by first mapping out all the connections between proximal ^1^H seen in the DQ/SQ spectrum, and then connecting each of the assigned ^1^H resonances to their attached carbon.

There is usually likely to be more spectral overlap in the ^1^H–^1^H DQ/SQ spectrum than in an analogous ^13^C–^13^C refocused INADEQUATE spectrum, and these two correlation spectra alone are often not sufficient. As a result the probabilistic assignment approach^244^ described above is used by default, and spectral editing methods can also be used as needed.

This ^1^H detected approach has recently been described in detail, for example, for the assignment of the ^1^H and ^13^C resonances in Verinurad.^128^ Currently, the ^1^H approach allows complete assignment in molecular solids with up to about 30 carbon atoms. The main limit of the ^1^H detected approach in molecular solids is poor ^1^H resolution. Due to the dense network of ^1^H–^1^H dipolar couplings, ^1^H spectra respond differently to MAS than dilute nuclei such as ^13^C or ^15^N, and ^1^H spectra get progressively narrower as spinning gets faster.^292–294^ At spinning rates up to around 60 kHz, additional narrowing can be obtained by using dipolar-decoupling pulse sequences in CRAMPS type approaches^236,295–298^ (and indeed this is what is usually done in *t*_1_ of the carbon-13 detected HETCOR experiments discussed above (Fig. 11e)). However, so far, no additional narrowing has been observed above 60–65 kHz MAS from CRAMPS type approaches, and the best ^1^H resolution obtained today by coherent averaging is from MAS spectra spinning at the fastest MAS rates available (∼160–180 kHz) in the highest magnetic fields,^254^ with good representative contemporary examples for molecular solids being the 800 MHz spectra of Verinurad at 160 kHz MAS in Fig. 13,^128^ or the 1 GHz spectra of Ritlectinib tosylate at 60 kHz MAS obtained by Rehman *et al.*^129^ Even with recent advances, typical ^1^H linewidths obtained at 100 kHz MAS still comprise 100–400 Hz of dipolar broadening.^293^

**Fig. 14:**
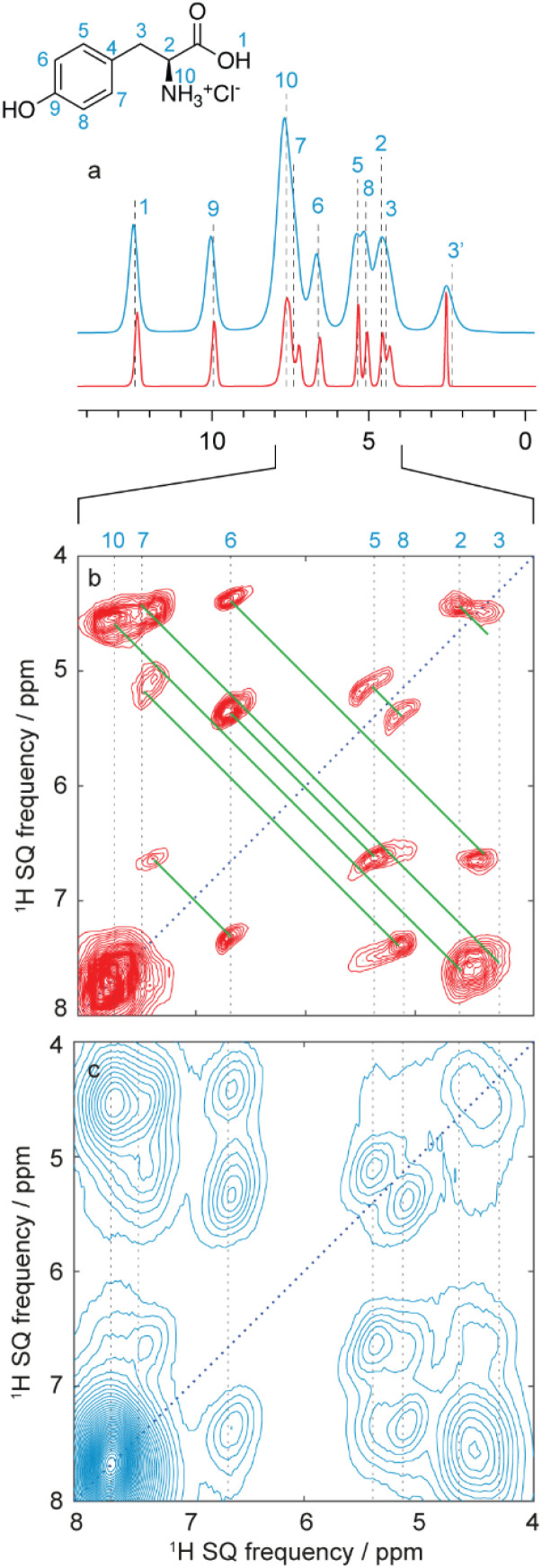
(a) Pure isotropic ^1^H spectrum of l-tyrosine hydrochloride obtained using a 2D dataset of 36 MAS spectra recorded at spinning rates from 30 to 100 kHz (red), as compared to the 100 kHz MAS spectrum (blue). The variable rate dataset was transformed using the PIPNet deep learning model.^300^ (b and c) Expansions of 100 kHz MAS 2D ^1^H–^1^H DQ/SQ BABA spectra (blue) and pure isotropic 2D ^1^H–^1^H DQ/SQ BABA spectra (red). The isotropic spectrum was inferred with the PINet2D model from a 3D VMAS dataset of 11 2D spectra recorded at MAS rates between 50 and 100 kHz.^301^ Both spectra are shown after shearing to an SQ/SQ representation. Vertical lines indicate the previously assigned proton shifts at 100 kHz MAS,^299,300^ and the green lines the observed double quantum correlations. Adapted from ref. 300 and 301.

An alternative approach to ^1^H line narrowing was recently introduced where instead of trying to optimize and perfect a coherent averaging scheme to minimize errors that cause residual dipolar broadening, the errors are mapped into a second dimension of a 2D correlation experiment.^299^ For example, in a dataset of ^1^H spectra acquired at different MAS rates, the isotropic shifts do not change as a function of MAS rate, but the dipolar contribution (broadening and shift) scales with the rate. These 2D datasets can be transformed using deep learning models to yield a one-dimensional pure-isotropic proton (PIP) spectrum.^300^ These new approaches provide the highest ^1^H NMR resolution available today in rigid solids. In analogy, a 3D dataset of (for example) ^1^H–^1^H DQ/SQ spectra acquired at different MAS rates can be transformed to obtain a 2D pure-isotropic ^1^H–^1^H DQ/SQ correlation spectrum.^301^ These methods were used in the assignment of Verinurad,^128^ and an example of the increased resolution this provides for the case of l-tyrosine hydrochloride is shown in Fig. 14.^300,301^ These new methods enable assignment of more crowded ^1^H spectra and open up new routes to ^1^H detected strategies.

## Conclusions

In summary, the development of new NMR methods, in combination with advanced computational methods, over the last 20 years has led to robust and broadly applicable methods for atomic-level structure determination in materials where structure and dynamics were previously the most inaccessible. These range from powdered micro-crystalline compounds to complex hierarchical hybrid materials.

The advent of these advanced NMR crystallography methods has been enabled in particular: by the introduction of new strategies for structure determination from chemical shifts through first principles calculations, augmented by machine learning and large scale data mining approaches; by new strategies for dynamic nuclear polarization enhanced NMR through innovative approaches to generating hyperpolarization; and by developments in multi-dimensional super-resolution ^1^H NMR experiments, through ultra-fast magic angle spinning and new approaches to error mapping.

We note that here we have centred the discussion on molecular solids, but that analogous chemical shift led structure determination methods are also broadly developed and implemented for inorganic materials, and detailed discussions and reviews can be found elsewhere.^5,23,34^

Also, in this article the focus has been exclusively on the NMR methods. As mentioned briefly in the introduction, it should go without saying that the NMR led methods here will always be used in combination with any other structural information available, whether from other methods (such as STM, TEM, PXRD, micro-ED…), or from other NMR probes.

The impact of these advances cannot be understated, as they provide the possibility for rational design of better properties in all the application areas discussed above.

## Data availability

There is no primary data associated with this article.

## Conflicts of interest

There are no conflicts of interest to declare.
